# Supplementary material for: New Faecal Calprotectin Assay by IDS: Validation and Comparison to DiaSorin Method
Source: Diagnostics (Basel). 2022 Sep 27;12(10):2338. doi: 10.3390/diagnostics12102338 (PMC9600005; doi:10.3390/diagnostics12102338)
Supplement: Supplementary file 1 [file diagnostics-12-02338-s001.zip › diagnostics-1908454-supplementary.pdf]

Table S1. user satisfaction form.

Criterion	Sensitivity	95% CI	Specificity	95% CI
>26µg/g	90.24	76.9 - 97.3	52.63	40.8 - 64.2
>132µg/g	65.85	49.4 - 79.9	86.84	77.1 - 93.5
>248µg/g	51.22	35.1 - 67.1	92.11	83.6 - 97.0
